# Conventional extraction of fucoidan from Irish brown seaweed *Fucus vesiculosus* followed by ultrasound-assisted depolymerization

**DOI:** 10.1038/s41598-024-55225-z

**Published:** 2024-03-14

**Authors:** Viruja Ummat, Saravana Periaswamy Sivagnanam, Dilip K. Rai, Colm O’Donnell, Gillian E. Conway, Shane M. Heffernan, Stephen Fitzpatrick, Henry Lyons, James Curtin, Brijesh Kumar Tiwari

**Affiliations:** 1 Teagasc Ashtown Food Research Centre, Teagasc, Ashtown, Dublin 15, Ireland; 2 UCD School of Biosystems and Food Engineering, University College Dublin, Belfield, Dublin 4, Ireland; 3 BiOrbic Bioeconomy SFI Research Centre, University College Dublin, Belfield, Dublin 4, Ireland; 4 Department of Biological Sciences, Munster Technological University, Bishopstown, T12P928 Cork Ireland; 5 In Vitro Toxicology Group, Institute of Life Science, College of Medicine, Swansea University, Swansea, Wales SA3 5AU UK; 6 Applied Sports Science Technology and Medicine Research Centre (A-STEM), Faculty of Science and Engineering, Swansea University, Swansea, Wales SA3 5AU UK; 7 Nutramara Ltd., Beechgrove House Strand Street, Tralee, Ireland; 8 School of Food Science and Environmental Health, College of Science and Health, Technological University Dublin, Dublin, D07 ADY7 Ireland

**Keywords:** Seaweed, Fucoidan, Extraction, Ultrasound, Depolymerization, Biochemistry, Green chemistry, Biotechnology

## Abstract

Fucoidan has attracted considerable attention from scientists and pharmaceutical companies due to its antioxidant, anticoagulant, anti-inflammatory, anti-tumor, and health-enhancing properties. However, the extraction of fucoidan from seaweeds often involves the use of harsh chemicals, which necessitates the search for alternative solvents. Additionally, the high viscosity and low cell permeability of high molecular weight (Mw) fucoidan can limit its effectiveness in drug action, while lower Mw fractions exhibit increased biological activity and are also utilized as dietary supplements. The study aimed to (1) extract fucoidan from the seaweed *Fucus vesiculosus* (FV) using an environmentally friendly solvent and compare it with the most commonly used extraction solvent, hydrochloric acid, and (2) assess the impact of ultrasound-assisted depolymerization on reducing the molecular weight of the fucoidan extracts and examine the cytotoxic effect of different molecular weight fractions. The findings indicated that the green depolymerization solvent, in conjunction with a brief ultrasound treatment, effectively reduced the molecular weight. Moreover, a significant decrease in cell viability was observed in selected samples, indicating potential anticancer properties. As a result, ultrasound was determined to be an effective method for depolymerizing crude fucoidan from *Fucus Vesiculosus* seaweed.

## Introduction

Fucoidans are heteropolysaccharides found in brown seaweeds, which mainly consist of fucose, uronic acids, and galactose, xylose, mannose, arabinose, glucose and sulphate groups. These along with their low Mw oligosaccharide derivatives have been investigated for their wide range of health benefits^1^. Fucoidan has a diverse structure, Mw and sulfation pattern, depending on tissue, growth-stage, geographic location, environmental conditions, season and the extraction process^2^. Many studies have reported on its biological activities such as anti-tumor, antioxidant, anticoagulant, antithrombotic, immunoregulatory, antiviral and anti-inflammatory effects^3^, which has led to a number of studies investigating development of new drugs and functional materials. However, the high viscosity, structural heterogeneity and low cell permeability of the high molecular mass polysaccharides, leads to low functionality in drug action^4^ and has hampered their development and application. Low Mw fucoidan fractions, which are a common form of fucoidan, have enhanced biological activity and are being used in food supplement and pharmaceutical product applications. The biological properties of fucoidan depend on a number of factors, including the relative abundance of sulfate groups, structural features, and molar mass distribution^5^. There is a lack of published data regarding variation of the fucoidan structure according to species, location, season and maturity and also, for identification of the optimum harvesting time and ensuring consistent product composition^6^.

For application, fucoidans must permeate the biological membranes to exert their effects in vivo and generally depolymerization of high molar mass compounds to fragments below 30 kDa results in more active fractions^5^. Therefore, depolymerization to obtain low Mw polysaccharides is recommended, to produce low molar mass oligosaccharides with improved physiological activities and novel biological effects^1^. For depolymerization of fucoidan, various methods have been employed such as chemical hydrolysis^7^, enzymatic method^8,9^, hydrothermal method^10–12^, Ultrasound (US) alone^13^ and with H_2_O_2_^4^, fucoidanase (Enzyme) degradation^14^, radical method^15^ and gamma irradiation^16^.

Amongst these approaches, US is an effective method of depolymerization, which has been used to depolymerize starch and research related to the same was first reported by^17^. Compared to chemical, thermal and enzymatic treatments, US offers a broad number of advantages such as, no requirement of any other substances, higher frequency of polymer breakage towards the middle of the chain, preventing formation of monomers and no side reactions. US treatment of samples simply required use of ultrasonic baths or immersion probes, without the need of complex and expensive set-ups^18^. Unlike irradiation, US is not strictly regulated, can be done anywhere and does not involve the use of potentially harmful materials.

US-assisted depolymerization, involves three effects^19^, namely primary effect including the processes occurring in gas phase inside the bubble; secondary effects involving the solution phase; and also physical effects caused by the shock waves arising from adiabatic collapse of a cavitation bubbles^20^. The author also mentions that US is a simple and effective method of polysaccharide depolymerization, however limited studies have been reported on the ultrasonic depolymerization of fucoidan.

The ultrasonic degradation of polysaccharides produces low Mw products that exhibit better antioxidant and anti-inflammatory activities compared to the original sample^21^. Cancer is a collection of diseases marked by the unregulated proliferation and dissemination of abnormal cells. Without intervention, these cells can inflict severe damage and may even lead to fatality.

Despite significant advancements in medical research, cancer remains one of the leading causes of death worldwide^22^. Glioblastoma (GBM), which represents about half of all primary malignant tumors in the central nervous system, is the most prevalent malignant brain tumor^23^. Despite undergoing aggressive treatments such as extensive surgical removal, chemotherapy, and radiation therapy, the response to treatment is often poor. It has one of the shortest survival rates amongst all cancers^24^, and represents the highest number of potential years of life lost among all prevalent human cancers^25^. A variety of cancer treatment drugs, such as anthracyclines, methotrexate, and folic acid analogues, have been explored. These drugs target cells that divide and grow rapidly, which are common characteristics of cancer cells. They also target mechanisms that are often disrupted within cancer cells. However, the harmful effects of these drugs on healthy cells limit the amount that can be administered, thereby affecting their effectiveness^26^. For Glioblastoma (GBM), the last significant progress was made in 2005 with the approval of Temozolamide (TMZ). It was discovered that when TMZ is combined with radiation therapy, the median survival rate increases from 12.1 months (with only radiation therapy) to 14.6 months. However, there has been no substantial improvement in survival rates over the past 15 years^27^.

Cancer refractory to existing therapies has led to an increase in research for new therapeutic treatments including those from natural sources, amongst which marine sources like seaweeds containing fucoidan (Mw: average 20,000) have been widely investigated.

Fucoidan from *Fucus vesiculosus* has shown inhibitory effects when studied on cell lines of different types of cancers such as breast cancer, B-cell lymphoma, T-cell lymphoma, fibroblastic sarcoma, uterine sarcoma, lung cancer, hepatocellular carcinoma, colorectal cancer, keratinocytes, melanoma, pancreatic cancer etc., as reviewed by Van Weelden et al.^28^.

Studies reporting the anticancer and anti-tumor activities of fucoidan from brown algae have been widely reviewed^22,29–31^. However, to further demonstrate fucoidan’s potential utility in cancer treatment, there is a need to develop a standardised purification method and reduce fucoidan Mw from *Fucus vesiculosus* (*F. vesiculosus)*. Studies demonstrating ultrasound assisted depolymerization have been reported by Bagale^32^ and Torres^33^.

Torres^33^ treated *Sargassum muticum* extracts with ultrasound (37 and 80 kHz) for up to 120 min, and later analyzed the fractions for phenolic content, antioxidant capacity, sulfate, oligosaccharide content, and cytotoxicity against human cervical carcinoma cells (HeLa 229). On the other hand, Bagale^32^ studied the impact of ultrasound process parameters on the molecular weight, structure, and antioxidant activity of fucoidan from *Fucus vesiculosus.* The authors reported that 33 °C, a sonication time of 56 min, and a sonication intensity of 116 W/cm^2^ were the ideal sonication treatment conditions. They also mentioned that the sonication treatment led to a decrease in molecular weight. However, the authors also noted that the treatment time was long and could lead to the breakage of the structure of fucoidan, resulting in further desulfonation. The impact on cell viability was not studied in this case. Hence, there is a need to study the impact of ultrasound as a depolymerization method for fucoidan from *Fucus vesiculosus* over a shorter period of time, and to evaluate its impact on cell cytotoxicity.

The objective of this study is to investigate (a) extraction of crude fucoidan from brown seaweed *F. vesiculosus* using 0.1 M HCl, green extraction solvent (confidential) and a commercially available fucoidan, and (b) US-assisted depolymerization of the crude fucoidan using three solvents (distilled water, 0.1% citric acid and Fenton reagent), and (c) effect of different Mw fucoidans on cell viability. The green extraction solvent used is an environmentally friendly extraction solvent and is suitable for consumption. However, since this solvent is used by industry collaborators, the details cannot be disclosed.

## Results and discussion

Conventional extraction was carried out using a GES and 0.1 M HCl to obtain crude fucoidan extracts from *Fucus vesiculosus.* The extract obtained contains additional biomolecules such as alginic acid, in addition to fucoidan, and is therefore referred to as crude fucoidan. Dried crude fucoidan samples were further treated with 20 kHz US (amplitude 40, 70 and 100%) for 30 min, using different depolymerization solvents (0.1% citric acid, Fenton and distilled water). The depolymerized fractions were subsequently studied for cell viability.

### Crude fucoidan

Fucoidan can be extracted from brown seaweeds by various multistage processes using chemical, physical and/ or enzymatic methods, while preserving native properties^19^. The extraction involves diffusion of solvent inside the solid matrix, hydrolysis/solubilisation of target compounds, diffusion of the compounds through the solid matrix and into the bulk solution.

Since the biomolecules are embedded deep in the matrices, it becomes important degrade cell walls, to facilitate the extraction^34^. Hot water and/ or acids (hydrochloric acid, sulphuric acid) or calcium chloride salt are commonly used for extraction of fucoidan^35^. Several factors including temperature, extraction time, pH, liquid–solid ratio and the number of stages involved, influence the extraction of fucoidan^36^. Solvent pH plays an important role in the extraction of fucoidan as it can enhance or hinder the yield of fucoidan obtained. For example, in industrial extraction of fucoidan from *Sargassum* sp., an increase in pH from 3 to 5 led to a significant increase in fucoidan yield, while a decrease was observed at pH 7. Also pH was found to show a significant interactive effect with temperature and buffer: alga ratio on fucoidan yield (P < 0.05).

**Figure 1:**
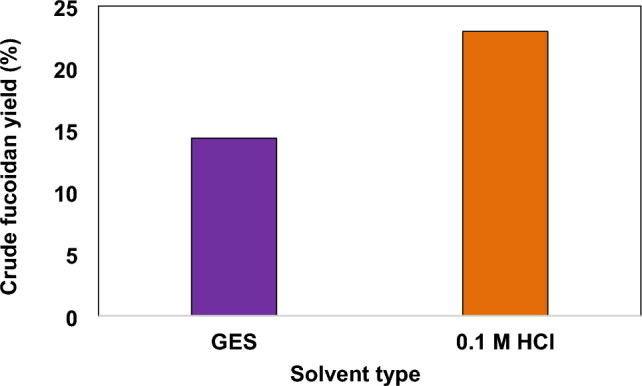
Crude fucoidan yield from *Fucus vesiculosus* obtained using GES and 0.1 M HCl.

It was inferred that low pH can lead to polysaccharide degradation, while high pH can coextract high quantities of alginates which may can increase the solution viscosity and interfere with extraction of fucoidan^37^. From our study crude fucoidan yields of 14.34% and 22.95% were obtained using GES and 0.1 M HCl respectively (Fig. 1). The results demonstrate that HCl resulted in a higher yield of crude fucoidan and is a more efficient extraction solvent compared to GES. Extraction of protein, amino acids and polysaccharides from seaweeds, depends on several factors including type of seaweed, extraction solvent and extraction time. The extraction of polysaccharides from seaweed depends on the solvents that can dissolve the algae cell components and interfere with the hydrogen linkages in the polysaccharide chains^38^.

To obtain fucoidan and alginates from the brown alga *Ecklonia radiata*^39^ performed an acid treatment with HCl solution. This method extracted fucoidan and also enabled the efficient sequential extraction of alginates. The authors reported that the acid disrupts the hydrogen bonds between polysaccharides and facilitates release of fucoidan. The H + of the acid (HCl) can do this more effectively than the GES, which has a lower acidity. Therefore, 0.1 M HCl is a better solvent than GES for extracting polysaccharides from seaweed, as it has a higher extraction yield. However due to safety concerns relating to HCl, edible-grade GES is a viable alternative for fucoidan extraction.

The findings are consistent with a previous study on brown seaweed *Sargassum fusiforme*^40^, where it was reported that 1 M HCl resulted in more than a two times higher polysaccharide yield compared to using water. Similar findings were reported for *Fucus virsoides* and *Cystoseira barbata*^41^, where 0.1 M HCl and 0.1 M H_2_SO_4_ resulted in higher polysaccharide yields from *Fucus virsoides* and *Cystoseira barbata* compared to using water.

### Depolymerization of fucoidan

The Mw of the crude fucoidan samples obtained using the GES and 0.1 M HCl were 399.99 ± 13.28 and 270.43 ± 14.88 kDa respectively. In comparison a Mw of 136.31 ± 5.02 was obtained for the reference commercial fucoidan product analysed. The effect of US treatments (amplitude 40, 70, 100%) and depolymerization solvents (distilled water, 0.1% citric acid and Fenton reagent) on fucoidan samples was analysed.

### Effect of ultrasound on Mw reduction of fucoidan samples

All US treatments investigated using selected depolymerization solvents reduced the Mw of fucoidan (Table 1). Differences in Mw of fucoidan samples, depolymerized with US using distilled water, 0.1% citric acid and Fenton reagent are depicted with superscripts a–g, h–o and p–u respectively. At higher US amplitudes increased levels of depolymerization were observed. The maximum reduction in Mw was observed for GES samples subjected to US treatment at 100% amplitude. These results are consistent with a previous study on the depolymerization of raw κ-carrageenan using US, where the authors reported that that high sonication amplitudes and longer treatment time resulted in higher Mw reduction^21^.

**Table 1:** Effect of ultrasound amplitude treatment using distilled water, 0.1% citric acid and Fenton reagent at 30 °C for 30 min on Mw of fucoidan samples.

Ultrasonic (US) amplitude	Depolymerization solvent	Molecular weight [average (kDa) + S.D.]		
		GES	0.1 M HCl	Commercial fucoidan
	Crude fucoidan (Native)	399.99 ± 13.28^b^	270.43 ± 14.88^e^	136.31 ± 5.02^g^
US 40%	Distilled water	365.51 ± 11.14^b^	186.98 ± 4.59^d^	133.84 ± 5.6^g^
US 70%	Distilled water	124.47 ± 3.45^a^	126.30 ± 0.86^c^	111.08 ± 3.99 f.
US 100%	Distilled water	119.61 ± 0.87^a^	111.19 ± 0.15^c^	104.67 ± 2.24^f^
	Crude fucoidan (Native)	399.99 ± 13.28^i^	270.43 ± 14.88^l^	136.31 ± 5.02^o^
US 40%	0.1% citric acid	384.98 ± 1.58^i^	206.30 ± 0.56^k^	124.09 ± 1.47^n^
US 70%	0.1% citric acid	134.48 ± 1.59^h^	116.57 ± 0.11^j^	109.45 ± 1.00^m^
US 100%	0.1% citric acid	117.98 ± 1.88^h^	107.49 ± 0.29^j^	105.99 ± 1.93^m^
	Crude fucoidan (Native)	399.99 ± 13.28^q^	270.43 ± 14.88^s^	136.31 ± 5.02^u^
US 40%	Fenton reagent	27.75 ± 0.82^p^	25.21 ± 0.38^r^	33.66 ± 0.20^t^
US 70%	Fenton reagent	26.75 ± 0.43^p^	20.89 ± 0.68^r^	27.24 ± 0.05^t^
US 100%	Fenton reagent	23.38 ± 1.18^p^	20.08 ± 0.51^r^	31.45 ± 0.17^t^

The results are expressed as average ± standard deviation. Statistical differences in Mw of fucoidan samples, depolymerized with US using distilled water, 0.1% citric acid and Fenton reagent are depicted with superscripts a–g, h–o and p–u respectively. *P* < 0.05.

Factors that influence the rate of polymer degradation during ultrasonic depolymerization include US intensity, treatment duration and solution concentration. Increasing US intensity generates more cavitation bubbles, which contribute to the degradation process. Ultrasonic degradation is a simple and effective method of polysaccharide depolymerisation, characterised by a high rate of decomposition of large Mw molecules. Cavitation bubbles are formed which can cause intense local heating and high pressure during their collapse. As a result of the bubble collapse, energy is released in the amount sufficient to break the chemical bonds in any polymeric materials.

The rupture of polymer chains as a result of sonolysis occurs in the middle of the molecule, with a greater effect when exposed to low-frequency ultrasound. Increasing US intensity generates more cavitation bubbles, which contribute to the degradation process^19^.

Tecson et al.^21^ reported that US was efficient in reducing the Mw of all raw κ-carrageenan samples, which can be attributed to the homolytic bond breaking and subsequent reaction with radicals facilitated by the high velocity gradients, temperature (up to 5000 °C), and pressure (about 5 × 10^7^ Pa) generated by collapsing cavitation bubbles. When polymers enter the high velocity gradient areas, they are stretched, distorted and stress is generated within the polymer resulting in their bond breakage. In a study involving US and, it was observed that an increase in ultrasonic intensity (424 W/cm^2^), led to larger reduction in Mw^42^. In another study on US-assisted depolymerization (30 and 80 kHz US frequencies, 30–180 min treatment time) of fucoidan from *Sargassum muticum*. The authors reported that high US treatment for long time promoted the depolymerization. Also, with 80 kHz, the phenolic content and antioxidant capacity were observed to be increased up to 120 min^43^.

The results are expressed as average ± standard deviation of the mean. The difference in Mw of fucoidan samples, depolymerized with US using distilled water, 0.1% citric acid and Fenton reagent are depicted with superscripts a–g, h–o and p–u respectively. The superscripts representing the impact of US for each solvent, indicate the difference in Mw for US treatments at amplitudes of 40%, 70%, and 100%. For example letters a and b represent the difference between GES crude sample and GES depolymerized sample obtained using distilled water and different ultrasonic amplitudes. P < 0.05.

### Effect of solvent on Mw reduction of fucoidan samples

The effect of selected solvent type on the depolymerization of crude fucoidan is shown in Table 2. It was found that depolymerization solvent and US treatment employed influenced the Mw reduction. The Fenton solvent resulted in the highest degree of depolymerization, followed by 0.1% citric acid and distilled water. It has also been reported that US treatment of beta-carotene using 21–25 kHz was limited in reducing the Mw of < 20 kDa due to energy transmission attenuation under a prolonged or high-intensity ultrasonic field^44^. To achieve greater depolymerization, the use of different solvents needs to be studied along with US treatment applied.

**Table 2:** Effect of depolymerization solvents using ultrasound treatments (40, 70 and 100% amplitude, 30 °C for 30 min) on Mw of fucoidan samples.

Ultrasonic (US) amplitude	Depolymerization solvent	Molecular weight [average (kDa) + S.D.]		
		GES	0.1 M HCl	Commercial fucoidan
Crude fucoidan	Crude fucoidan (Native)	399.99 ± 13.28B	270.43 ± 14.88E	136.31 ± 5.02G
US 40%	Distilled water	B	D	G
US 40%	0.1% citric acid	B	D	G
US 40%	Fenton reagent	A	C	F
Crude fucoidan	Crude fucoidan (Native)	399.99 ± 13.28J	270.43 ± 14.88M	136.31 ± 5.02P
US 70%	Distilled water	I	L	O
US 70%	0.1% citric acid	I	L	O
US 70%	Fenton reagent	H	K	N
Crude fucoidan	Crude fucoidan (Native)	399.99 ± 13.28S	270.43 ± 14.88V	136.31 ± 5.02Y
US 100%	Distilled water	R	U	X
US 100%	0.1% citric acid	R	U	X
US 100%	Fenton reagent	Q	T	W

The results are expressed as average ± standard deviation. Different letters indicate statistical differences in the Mw of fucoidan samples depolymerized using depolymerization solvents at 40%, 70% and 100% US amplitude. The uppercase letters representing the impact of solvent for each US amplitude. For example, difference between Mw of GES crude fucoidan and GES samples obtained with 40% US amplitude and different solvents are labelled as A–B. P < 0.05.

US-assisted of pectin has also been studied for pectin. For example, Zhi et al.^45^ investigated the depolymerization of pectin using US at 22 kHz and Fenton to produce ultra-low Mw pectin. Their US- Fenton process reduced the 448.26 kDa Mw of pectin to 53.52 kDa in 5 min, and after 35 min the Mw was reduced to 5.5 kDa. In another study^42^, observed that US even in combination with H_2_O_2_ failed to degrade the sulfated polysaccharide from sea cucumber *Isostichopus badionotus* (fCs-Ib) into low Mw fragments within 90 min. The authors suggested that H_2_O_2_/ascorbic acid system might be a more efficient way to prepare low Mw fCS-Ib.

Li et al.^46^ prepared rhamnogalacturonon-I enriched low Mw pectic polysaccharide using US (22 kHz, 900 W) and metal free Fenton. They observed that there was a higher reduction in Mw using US/H_2_O_2_/ascorbic compared to H_2_O_2_/ascorbic without US. This shows the synergistic effect of US and the solvent used. However the use of hydrogen peroxide in food industry is limited by regulations in some countries.

In a study reported by Zheng et al.^47^, the authors investigated the impact of US (120 W), hydrogen peroxide concentrations (0.5, 1, 1.2, 1.5%) and 30, 60, 90, 120, 150 and 180 min treatment time on degradation of chitosan. The results indicated that an increase in hydrogen peroxide concentration and US exposure time led to an increase in chitosan degradation.

### Cytotoxicity of fucoidan

**Figure 2:**
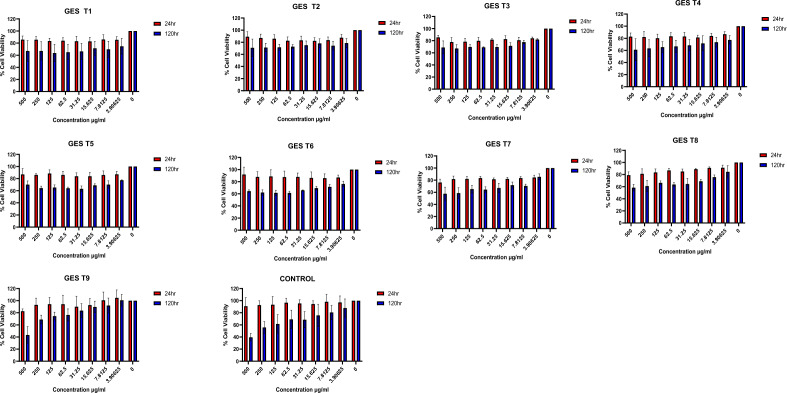
Cell viability assay for fucoidan obtained using GES and depolymerized using US and selected depolymerization solvents. Notation: GES T1—40% US with distilled water; GES T2—70% US with distilled water; GES T3—100% US with distilled water; GES T4—40% US with 0.1% citric acid; GES T5—70% US with 0.1% citric acid; GES T6—100% US with 0.1% citric acid; GES T7—40% US with Fenton; GES T8—70% US with Fenton; GES T9—100% US with Fenton, Control—Untreated samples (no fucoidan added). Mean ± standard deviation (n = 3).

**Figure 3:**
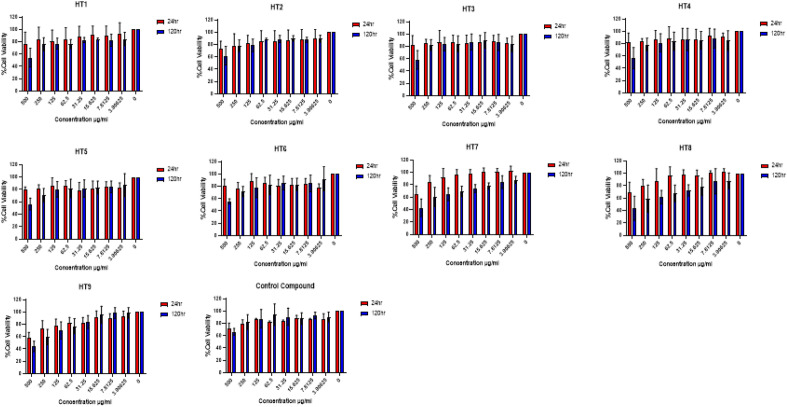
Cell viability assay for fucoidan obtained using 0.1 M HCl and depolymerized using US and selected depolymerization solvents. Notation: HT1—40% US with distilled water; HT2—70% US with distilled water; HT3—100% US with distilled water; HT4—40% US with 0.1% citric acid; HT5—70% US with 0.1% citric acid; HT6—100% US with 0.1% citric acid; HT7—40% US with Fenton; HT8—70% US with Fenton; HT9—100% US with Fenton; Control—Untreated samples (no fucoidan added). Mean ± standard deviation (n = 3).

**Figure 4:**
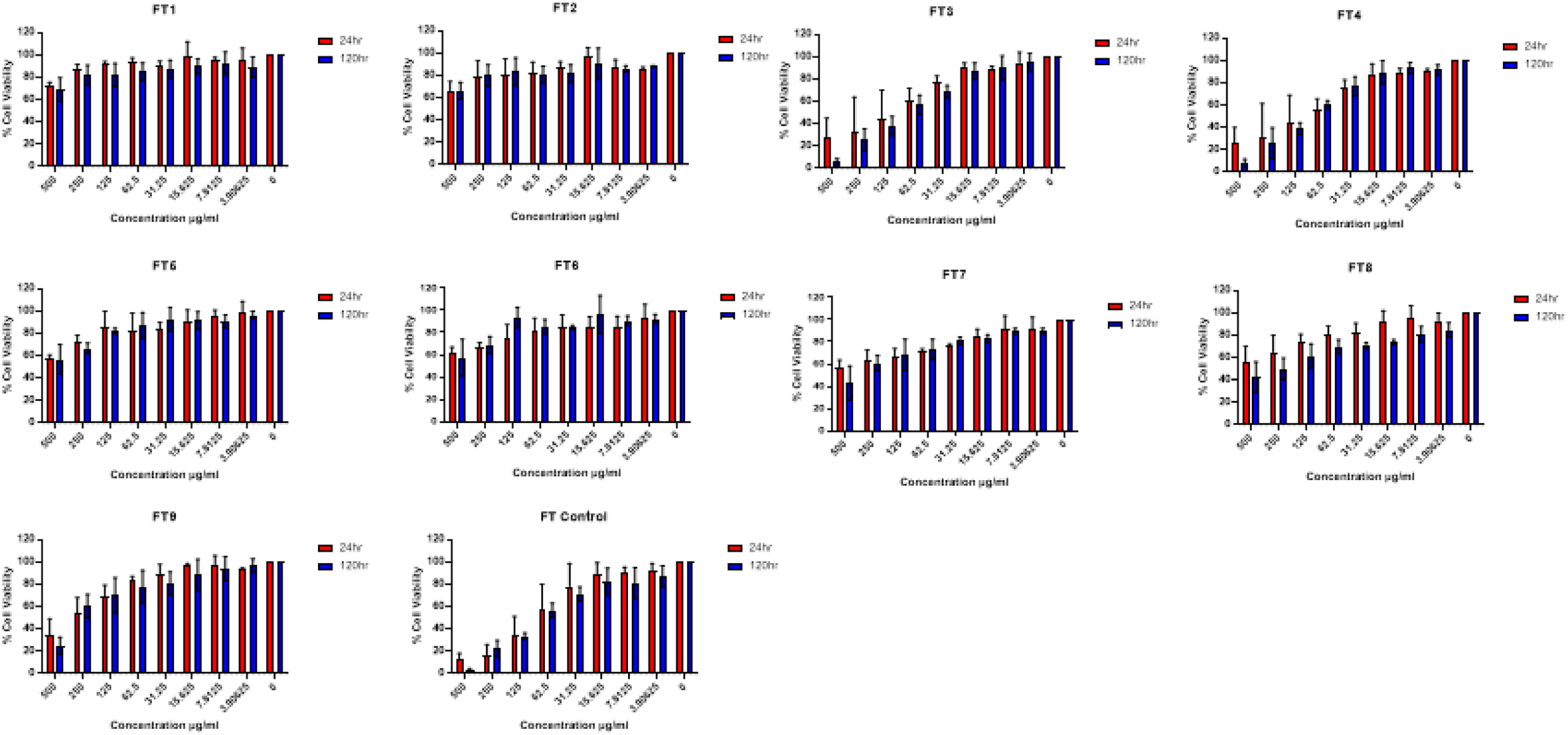
Cell viability assay for commercial fucoidan (labelled as F) depolymerized using US and depolymerization solvent. Notation: FT1—40% US with distilled water; FT2—70% US with distilled water; FT3—100% US with distilled water; FT4—40% US with 0.1% citric acid; FT5—70% US with 0.1% citric acid; FT6—100% US with 0.1% citric acid; FT7—40% US with Fenton; FT8—70% US with Fenton; FT9—100% US with Fenton; FT Control—Untreated samples (no fucoidan added). Mean ± standard deviation (n = 3).

**Figure 5:**
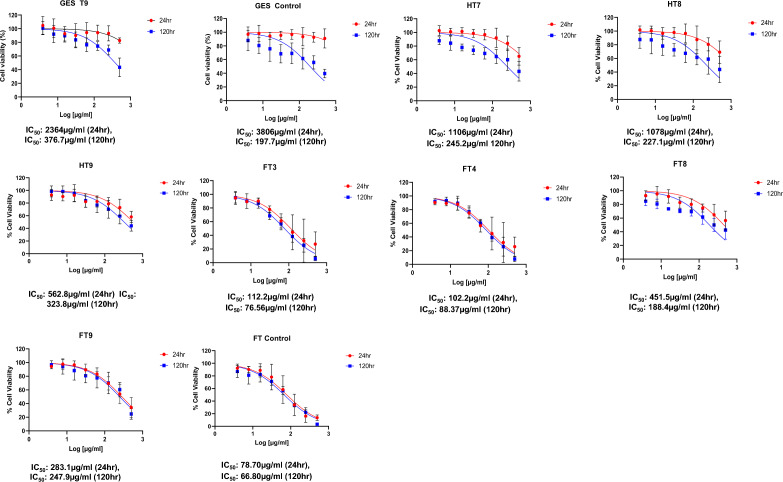
Non-linear regression and IC_50_ values for compounds demonstrating a dose response following 24 and 120 h exposure for i.e. fucoidan obtained using GES, 0.1 M HCl (H) and commercial fucoidan (F) and US depolymerization treatments: T3—100% US, distilled water; T4—40% US, 0.1% citric acid; T7—40% US, Fenton; T8—70% US, Fenton; T9—100% US; Fenton. Control—untreated samples (no fucoidan added).

Higher cytotoxicity activity was observed at higher fucoidan concentrations (Figs. 2, 3, 4). Lower Mw fucoidan samples exhibited higher cytotoxic effects against glioblastoma cells. Depolymerised fucoidan samples which showed a good dose response were analysed using regression analysis to generate IC_50_ values (Fig. 5). Li et al.^42^ reported that both native and depolymerized fucoidan chondroitin sulfate (fCs-Ib) samples inhibited the viability of A549 lung cancer cells. They reported that US treated low Mw samples showed higher anti-tumour activity than the native (fCs-Ib). Yang et al.^48^ reported that the biological activities of fucoidans are closely linked to their Mw and sulfate content. Several studies have reported that low-molecular-weight fucoidan (LMWF) is more biologically active than high-molecular-weight fucoidans (HMWF). However, LMWF obtained from acidic hydrolysis leads to reduced bioactivities due to the removal of sulfate groups. Therefore, degrading HMWF into LMWF without removing its functional groups is critical^31^.

Cho et al.^49^ studied the effects of sulfation levels in low and high Mw fucoidan compounds on in vitro anti-cancer activity using human stomach cancer cell line AGS. Fucoidan was partially hydrolysed under mild acid conditions to yield low Mw fucoidan, which was then fractionated by membrane ultrafiltration. High (> 30 kDa) and low (5–30 kDa) Mw fucoidan were observed to be over sulphated. They also found that their over sulphated fucoidan displayed higher (15–30%) anti-cancer activity They reported that the dose dependent antiproliferative activity of the 5–30 kDa Mw fraction against the stomach cancer cell line AGS was two times higher than that of the > 30 kDa fraction.

Cabral et al.^50^ investigated the influence of Mw fractionation on the antimicrobial and anticancer properties of a fucoidan rich-extract from the macroalgae *Fucus vesiculosus.* They reported that fucoidan fractions obtained using multiple Mw cut-off (MWCO) have potential to be used as natural and green bacteriostatic and bactericidal ingredients in the food industries.

Fractions that were extracted (> 300 kDa, < 300 kDa, < 100 kDa, < 50 kDa and < 10 kDa) were assessed for their cytotoxic effects on a U-251MG glioblastoma multiforme cancer cell line using the Alamar Blue assay over incubation periods of 24 h, 48 h, and 6 days. The > 300 kDa fraction demonstrated the lowest IC_50_ value against the tumoral cells compared to the other four fractions tested, with values ranging from 0.052% (24-h treatment) to 0.032% (6-day treatment). This was attributed to the increased sensitivity of cancer cells to fucoidan, glucans, and other anticancer polysaccharides present in the > 300 kDa fraction.

## Discussions

The potential of using green extraction solvent and 0.1 M HCl to extract crude fucoidan from brown seaweed *Fucus vesiculosus* was demonstrated*.* Due to safety concerns relating to HCl, edible grade GES is a viable alternative for fucoidan extraction. The method described in this chapter demonstrated a high efficiency in extracting fucoidan from seaweed biomass. This extraction method has a great potential for industrial applications, as it can be scaled-up to produce large quantities of fucoidan. The industry collaborator, Nutramara, uses this method for their commercial production of fucoidan. US treatment at the three amplitudes investigated was effective in depolymerizing high Mw crude fucoidan into low Mw samples for all solvents used. Samples with lower Mw exhibited higher cytotoxic effects compared to high Mw fucoidan samples. This study validated the importance of a suitable solvent along with US for efficient polymer degradation and demonstrated that US can be recommended as an efficient depolymerization method to obtain low Mw fucoidan from *Fucus vesiculosus.*

## Materials and methods

### Biological material

Seaweed *Fucus vesiculosus* was harvested from Galway Bay off the coast of Connemara, Co Galway. Collection of plant material was done by Nutramara Ltd, Kerry, Ireland and was in compliance with the national and EU regulations. Seaweeds were harvested and provided as per the licence agreement and Nutramara has the necessary licence/permissions to operate in Ireland. *Fucus vesiculosus* samples employed in the study are harvested commercially. Details of the samples are available publicly at National Biodiversity Data Centre, Ireland available online https://maps.biodiversityireland.ie/Species/187328. The formal identification of the seaweed was undertaken by Dr. Henry Lyons, Scientific Director-Nutramara Ltd., Tralee, County Kerry, Ireland. The samples were dried using an oven dryer at 50–60 °C over 48 h and milled using a hammer mill. Commercially available fucoidan from *Fucus vesiculosus* was also provided by Nutramara.

### Chemical reagents

The chemicals used were calcium chloride (Sigma-Aldrich), food-grade citric acid (Sigma-Aldrich), ethanol (EMPROVE exp Ph Eur, BP, JP, USP), 37% HCl (VWR BDH chemicals), L-Ascorbic acid (Sigma Aldrich), hydrogen peroxide (30% pure Ph. Eur., USP, Panreac A0626,1000) and NaCl (ACS reagent, ≥ 99%, Sigma-Aldrich).

### Conventional extraction of fucoidan and ultrasound-assisted depolymerization

**Figure 6:**
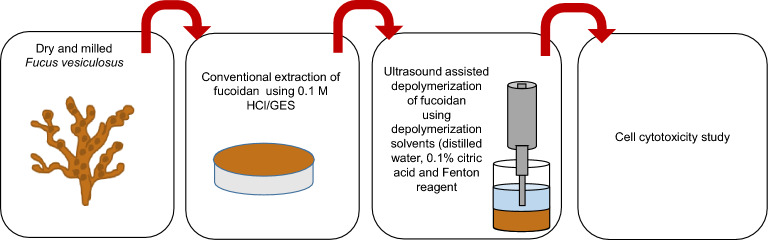
Process flow chart of fucoidan extraction and depolymerization.

The seaweed to solvent ratio was kept as 1/10 (w/ v). A green extraction solvent (GES) (pH 3.5) and 0.1 M HCl were used as solvents and conventional extraction at 80 °C, 200 rpm for 2 h was carried out. The seaweed and solvent mixture were kept in a water bath (85 °C) (Clifton range NE1–2.5 unstirred thermostatic bath, UK) and an overhead stirrer (VWR VOS 40 digital) was used to stir the mixture throughout the extraction process (Fig. 6). After 2 h, the mixture was cooled at room temperature and then filtered using a muslin cloth, to separate the residue and the supernatant. The supernatant was mixed with 1% (w/v) calcium chloride and stored at 4 °C. After 24 h, the mixture was centrifuged (3500 rpm, 15 min, 4 °C) using (Sorvall Lynx 6000 centrifuge, Waltham, MA, USA) and the pellets and supernatant were separated. The supernatant was then mixed with 1:3 ethanol (v/v) and stored for 24 h at 4 °C, followed by centrifugation (3500 rpm, 15 min, 4 °C). All the samples (pellets and supernatant) were freeze-dried, at 0.5 mbar, for 2 days using a freeze dryer (Lyovapor™, L-300, Buchi, Flawil, Switzerland).

The crude fucoidan extraction yield was determined by Eq. (1)

1 $$\mathrm{Crude \, fucoidan \, yield}(\mathrm{\%}) =\frac{\mathrm{Weight \, of \, dry \, extract }\times 100 }{\mathrm{Weight \, of \, dry \, sample}}$$

The fucoidan obtained after extraction was subjected to US treatment involving depolymerization solvents (0.1% citric acid, distilled water and Fenton reagent). A Fenton reagent was prepared using 48 mM ascorbic acid and 200 mM hydrogen peroxide solution^40^. Slight modifications were made, along with 40 mL of distilled water, 30 mL of 48 mM ascorbic acid and 30 mL of 200 mM hydrogen peroxide solution was used as the Fenton system. Crude fucoidan (3 g) was mixed with 100 mL depolymerization solvent and US treatments of 20 kHz, amplitudes of 40, 70 and 100% and treatment time of 30 min using US immersion probe, 20 kHz (UIP500hdT, Hielscher Ultrasonics GmbH, Teltow, Germany) were carried out. The temperature was controlled using a circulation water bath at 30 °C. After the treatments, the sample were freeze-dried and stored at 4 °C under dark conditions prior to further analysis.

### Fucoidan molecular weight determination

High performance liquid chromatography coupled with refractive index (HPLC-RI) detector was used to determine the Mw distribution. Fucoidan Mw was quantified using a HPLC system (Agilent 1200 LC system, Agilent Technologies, Santa Clara, California, USA) fitted with a refractive index detector connected to a guard column (OHpak SB-G 6B, 8 × 50 mm) and a Shodex OHpak SB-804 HQ with 6% cross-linked HPLC carbohydrate column of dimensions 8 mm × 300 mm (length × I.D.) (Shodex, Japan)^50^. Samples at a concentration of 2 mg/mL were prepared using the 0.1% NaCl and filtered through 0.45 µm PTFE filters (Econo Filter, Agilent Technologies) and 20 µL were injected into the column using an auto sampler. Separation was achieved using 0.1% NaCl at a constant flow rate of 0.5 mL/min. for 40 min at 40 °C.

Mw determination was performed by comparison of the retention times with those of pullulan standard from Sigma (Set Mp ~ 350–700,000, Sigma-Aldrich, St. Louis, MO, USA). The integration of the peaks was performed using the software Agilent Chemstation. A standard curve was developed using different Mw of pullulan. All analysis were performed in duplicate.

### Anti-cancer properties

The PrestoBlue cell viability assay was used to assess the cell viability at specific time points post treatment. The PrestoBlue cell viability assay (Thermo Fisher)^51^ involves a cell permeable resazurin based solution, which changes colour and becomes fluorescent with the reducing power of living cells, the change is detected by using absorbance or fluorescence measurements.

#### Cell culture

Patient derived glioblastoma cell lines were established by the Glioma Cellular Genetics Resource (gcgr.org.uk) funded by Cancer Research UK (Pollard et. al. Cell Stem Cell. 2009 4:568–80). The human glioma stem cell line, GCGR-E17, was cultured in DMEM/HAMS-F12 (ThermoFisher) supplemented with 1.5 g/L d-(+)-Glucose (Sigma), MEM non-essential amino acids (Gibco), Penicillin Streptomycin (Gibco), BSA (Gibco), 0.1 mM β-mercaptoethanol (Gibco), B27 (Gibco) and N2 supplements (Gibco). Cells were detached using accutase solution (Sigma) and sub-cultured every 5–7 days depending on confluency. Cell culture media was supplemented with 10 ng/mL mouse EGF (Preprotech), 10 ng/mL human FGF (Preprotech) and 1 μg/mL Laminin (Sigma).

#### Cell viability assay

96 well plates were coated with 10 µg/mL laminin for 24 h prior to use. The laminin coating was removed and cells plated at 1 × 10^4^ cells per well and left to adhere for 48 h. Fucoidan extracts were reconstituted in fresh media and filter sterilised using a 0.22 µM filter. GCGR-E17 cells were treated with decreasing concentrations from 500 to 0 µg/ mL of fucoidan extracts for 24 h or 5 days.

At the appropriate time point, cell media was removed from each well and replaced with 10% solution of Presto Blue cell viability reagent as per manufactures instructions. Fluorescence was measured with an automated microplate fluorometer (FLUOstar Omega, BMG LabTech) using an excitation wavelength of 544 nm and an emission wavelength of 590 nm. Cell viability was calculated as a percentage of the untreated control. Positive control for the viability assay for 24 h was H_2_0_2_ 1 mM (24 h) and TMZ 1 mM (5 days). Samples that were untreated were labelled as control.

### Statistical analysis

Data was analysed using SPSS version 27 (IBM SPSS Statistics). The treatments were compared with the crude samples and P < 0.05 was used for significance. One-way ANOVA and Tukey test were used to determine the difference. For cytotoxicity, nonlinear regression analysis was carried out using GraphPad Prism V9.

## Data Availability

The data can be obtained from the corresponding author on reasonable request.
